# CESAR: conventional ventilatory support vs extracorporeal membrane oxygenation for severe adult respiratory failure

**DOI:** 10.1186/1472-6963-6-163

**Published:** 2006-12-23

**Authors:** Giles J Peek, Felicity Clemens, Diana Elbourne, Richard Firmin, Pollyanna Hardy, Clare Hibbert, Hilliary Killer, Miranda Mugford, Mariamma Thalanany, Ravin Tiruvoipati, Ann Truesdale, Andrew Wilson

**Affiliations:** 1 Department of Cardiothoracic Surgery, Glenfield Hospital, Groby Road, Leicester, LE3 9QP, UK; 2 Medical Statistics Unit, London School of Hygiene and Tropical Medicine, Keppel St, London WC1E 7HT, UK; 3 Clinical Epidemiology and Biostatistics Unit, Royal Children's Hospital, Melbourne, Australia; 4 School of Medicine Health Policy and Practice, University of East Anglia, Norwich, NR4 7TJ, UK; 5 School of Health and Related Research, University of Sheffield and RTI Health Solutions, Williams House Manchester Science Park, Manchester ME15 6SE, UK; 6 Department of Health Sciences, University of Leicester, Leicester General Hospital, Leicester, LE5 4PW, UK

## Abstract

**Background:**

An estimated 350 adults develop severe, but potentially reversible respiratory failure in the UK annually. Current management uses intermittent positive pressure ventilation, but barotrauma, volutrauma and oxygen toxicity can prevent lung recovery. An alternative treatment, extracorporeal membrane oxygenation, uses cardio-pulmonary bypass technology to temporarily provide gas exchange, allowing ventilator settings to be reduced. While extracorporeal membrane oxygenation is proven to result in improved outcome when compared to conventional ventilation in neonates with severe respiratory failure, there is currently no good evidence from randomised controlled trials to compare these managements for important clinical outcomes in adults, although evidence from case series is promising.

**Methods/Design:**

The aim of the randomised controlled trial of **Conventional ventilatory support vs extracorporeal membrane oxygenation for severe adult respiratory failure (CESAR) **is to assess whether, for patients with severe, but potentially reversible, respiratory failure, extracorporeal membrane oxygenation will increase the rate of survival without severe disability ('confined to bed' and 'unable to wash or dress') by six months post-randomisation, and be cost effective from the viewpoints of the NHS and society, compared to conventional ventilatory support. Following assent from a relative, adults (18–65 years) with severe, but potentially reversible, respiratory failure (Murray score ≥ 3.0 or hypercapnea with pH < 7.2) will be randomised for consideration of extracorporeal membrane oxygenation at Glenfield Hospital, Leicester or continuing conventional care in a centre providing a high standard of conventional treatment. The central randomisation service will minimise by type of conventional treatment centre, age, duration of high pressure ventilation, hypoxia/hypercapnea, diagnosis and number of organs failed, to ensure balance in key prognostic variables. Extracorporeal membrane oxygenation will not be available for patients meeting entry criteria outside the trial. 180 patients will be recruited to have 80% power to be able to detect a one third reduction in the primary outcome from 65% at 5% level of statistical significance (2-sided test). Secondary outcomes include patient morbidity and health status at 6 months.

**Discussion:**

Analysis will be based on intention to treat. A concurrent economic evaluation will also be performed to compare the costs and outcomes of both treatments.

## Background

It is estimated that over 350 adult patients suffer from severe, but potentially reversible, respiratory failure in the UK each year. The mortality rate for such patients is very high and has only improved marginally in the majority of centres over the last 20 years[1,2] Current management uses intermittent positive pressure ventilation (IPPV). The airway pressures and oxygen concentrations required to maintain adequate blood gases are often very high in patients with severe respiratory failure, and this combination of barotrauma, volutrauma and oxygen toxicity can prevent lung recovery. An alternative treatment, extracorporeal membrane oxygenation (ECMO), uses cardio-pulmonary bypass technology to temporarily provide gas exchange to patients with severe, but potentially reversible, respiratory failure. During ECMO, ventilator settings can be reduced, and such 'lung-rest' allows the lungs to recover. There is currently no good evidence from randomised controlled trials (RCTs) to compare ECMO against conventional management for important clinical outcomes.

Patients are usually considered for ECMO when they have such severe disease that they continue to deteriorate despite maximal optimum 'conventional' treatment. For the purposes of this protocol, conventional will be defined as any treatment which relies on the patient's lungs to provide gas exchange. Conventional treatment may therefore include inhaled nitric oxide and prone ventilation[3-5], as well as the more usual types of positive pressure ventilation. The use of ECMO to support *neonatal *patients with severe respiratory failure has been rigorously evaluated in an RCT[6,7]. The neonatal ECMO RCT convincingly demonstrated the effectiveness of ECMO in improving patient survival without severe disability. Neonatal ECMO in the UK is now a supra-regional service receiving central funding. The use of ECMO as it is currently practised in older children[8], and adults[9] is more controversial, and has yet to be evaluated in an RCT in the UK.

### Previous studies

A review of the literature was carried out to identify all studies relevant to adult ECMO. Only two RCTs have been reported[1,10], both in the United States but they used such different approaches that they have not been combined as a formal meta-analysis. Each is detailed below, followed by the recent non-experimental evidence.

An RCT of adult ECMO was conducted by the US National Institutes of Health (NIH)[1], in the early days of extra-corporeal support in the 1970s. Survival in both groups was very poor (around 10%), and no difference was shown in survival between the conventional and ECMO treated groups. There were a number of important differences in the perfusion and ventilation techniques used during this trial compared to those used today. Firstly, veno-arterial (VA) rather than veno-venous (VV) perfusion was used, and this was thought to be responsible for the high incidence of pulmonary micro-thrombosis and fibrosis seen in the lungs of the ECMO patients (due to reduced pulmonary blood flow). Secondly, patients were anti-coagulated to such a degree that severe bleeding occurred. Thirdly, high pressure ventilation was continued during ECMO resulting in continued barotrauma and volutrauma[11,12]. Finally, the mean duration of ventilation prior to ECMO in the NIH ECMO trial was over 9 days, whereas it is now well-recognised that after 7 days of high pressure ventilation with high fraction of inspired oxygen (FIO_2_) the lungs only have limited powers of recovery[13].

More recently there has been an RCT of the related technique of extra-corporeal carbon dioxide removal (ECCO_2_R)[10]. This showed no difference between ECCO_2_R and conventional treatment. Again there were numerous differences in the clinical and perfusion protocols between this trial and those in widespread use in the majority of centres currently[14]. Firstly, the experimental arm of the trial used low flow ECCO_2_R in a group of patients who had severe lung disease, which warranted higher flow ECMO with full support of oxygenation and carbon-dioxide removal. This was demonstrated by the need to increase the airway pressure in the ECCO_2_R group half-way through the study. The reliance on the patient's lungs to provide oxygenation, especially at such high airway pressures, also eliminated any possibility for lung rest. Also, despite the involvement of one of the team in the 1970s NIH ECMO trial, in which VA ECMO was used with very small numbers in each centre (<5), the ECCO_2_R programme in this trial was not well developed prior to the study (as the team had only provided ECCO_2_R to sheep and one patient prior to starting the trial). The high incidence of bleeding and thrombotic complications reported in this study may attest to this inexperience. In addition, the conventional treatment used in the trial was Pressure Controlled Inverse Ratio Ventilation (PCIRV) using a computer controlled algorithm. The results of this treatment showed 44% survival compared to expected survivals of < 20% in other similar series of patients[2]. Despite this, survival in the ECCO_2_R group was the same as the 'conventional' group. The success of the PCIRV protocol in this study has led to the wide adoption of the technique within 'conventional' ventilatory management with survival of 66% for patients with *moderate to severe *respiratory failure (mean Murray score 2.8, mean ratio between the oxygen tension in the arterial blood and the fraction of inspired oxygen (PaO_2_/FIO_2_) 88 mmHg)[15]. Unfortunately no other authors have been able to duplicate the PCIRV results of Morris et al. for patients with *severe progressive *respiratory failure.

Because the two trials described above have little relevance to the ECMO regimens used in the majority of centres worldwide, the only relevant evidence consists of observational studies. By the nature of their design, the information they provide is potentially biased, and must therefore be viewed with caution.

Recent case series of patients with similar degrees of respiratory failure to the eligibility criteria for the second trial suggest survival rates without ECMO of 18% to 44%[1,10]. compared to rates of up to 66% with high flow ECMO (including full support of oxygenation and lung rest), provided by experienced teams principally in the USA, UK and Germany[9,13,14].

In a cohort study of the first 50 adult patients to receive ECMO for respiratory support at Glenfield Hospital, Leicester, UK, patients had severe respiratory failure as shown by the mean pre-ECMO Murray Lung Injury Score of 3.4 (SD 0.5) and PaO_2_/FIO_2 _ratio of 65 mmHg (SD 36.9). They were referred for ECMO with severe respiratory failure caused by either the Acute Respiratory Distress Syndrome (ARDS) or with pneumonia. The overall survival rate was 66%[9].

For the reasons outlined above, it is impossible to reach firm conclusions from the above experimental and observational data regarding the clinical effectiveness or cost-effectiveness of VV high flow ECMO for respiratory failure in adults. The recent evidence from observational studies does, however, suggest that ECMO could potentially be a highly useful treatment in these patients. The case selection and treatment protocols used during ECMO are now well defined by the international Extracorporeal Life Support Organization (ELSO), and the only team using ECMO in adults consistently in the UK has built up clinical expertise[9].

It is not possible to further define the safety and efficacy of ECMO as a treatment without a rigorous trial. The procedure has received a Cii categorisation (safety and/or efficacy not yet fully established; procedure requires a fully controlled evaluation) from the UK Safety and Efficacy Register of the New Interventional Procedures of the Medical Royal Colleges (SERNIP). Additionally a situation of equipoise currently exists, whereby clinicians can see the potential benefits of ECMO, but do not have enough evidence to make an informed choice as to the best treatment for their patient.

The aim of the present trial is therefore to assess whether for patients with severe, but potentially reversible, respiratory failure, ECMO will increase the rate of survival without severe disability by six months post randomisation and will be cost effective from the viewpoints of the NHS and society, compared to conventional ventilatory support.

## Methods/Design

### Design

**Figure 1:**
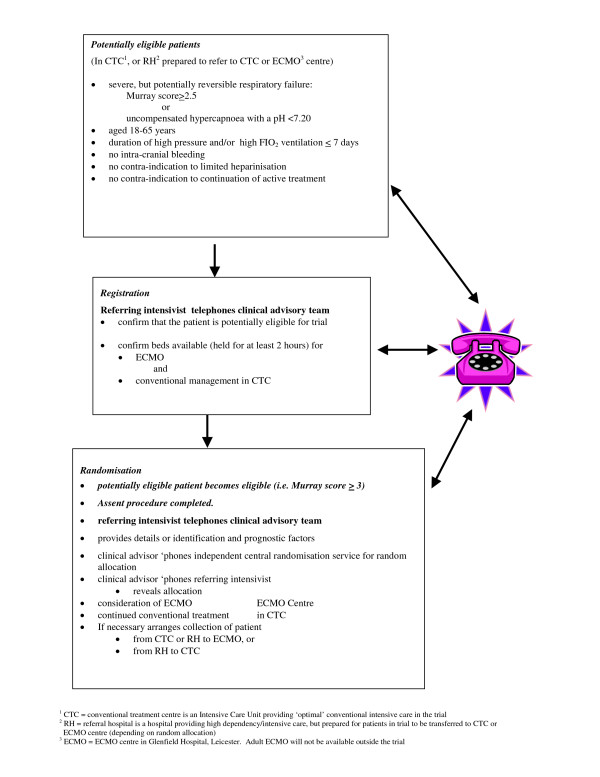
organisation of the trial.

The most scientifically rigorous design to assess effects of health interventions is that of an RCT. The design will be similar to the highly successful UK neonatal ECMO RCT[6] suitably adapted for the adult population. The design will be 'pragmatic' ie it will, as far as possible, mirror usual practice in the UK. The procedures are illustrated schematically in the Figure 1 below, and detailed in the text.

#### Primary hypotheses

The primary hypotheses are that, for patients with severe, but potentially reversible, respiratory failure, ECMO:

(a) Will increase the rate of survival without severe disability by six months post-randomisation.

(b) Will be cost effective from the viewpoints of the NHS and society, compared to conventional ventilatory support.

#### Inclusion criteria

##### i) Centres

(a) ECMO: This will be provided in the Glenfield Hospital, Leicester, which has 17 years of experience and is the only ELSO-recognised adult ECMO centre in the UK.

(b) Conventional treatment centres (CTC): These are either centres acknowledged by Critical Care Network leads (where established) to provide an appropriately high standard of conventional care for ECMO-eligible patients, or they are units which treat ≥ 350 patients per year, and can provide pressure controlled ventilation and veno-venous haemofiltration.

(c) Referral hospitals (RH): In addition to the centres described under (b) above, patients meeting ECMO entry criteria may be entered into the trial from other hospitals, if these hospitals are prepared to transfer the patient to a designated CTC should the allocation be to conventional management.

##### ii) Patients

Adult patients (18–65 years) with severe, but *potentially reversible *respiratory failure. Severe respiratory failure will be defined as a Murray score (appendix 1)[16] ≥3.0, or uncompensated hypercapnea with a pH <7.20. This level of hypercapnea was selected to reflect common intensive care clinical practice. The Murray score must be calculated using all 4 parameters (PaO_2_/FIO_2_, Positive End Expiratory Pressure (PEEP), Lung compliance and Chest X-ray appearance). The Murray score of 3.0 is a MINIMUM entry criterion. Since patients may deteriorate quickly and conventional treatment must be optimised prior to referral into the trial, intensivists will also have the option to discuss registration of the patient for the trial as soon as the Murray score exceeds 2.5. If the patient then continues to deteriorate, prior identification of available beds, and discussion of the trial with the relatives, will allow rapid randomisation and trial entry.

### Exclusion criteria prior to trial entry

• Duration of high pressure (> 30 cm H_2_O of peak inspiratory pressure) and/or high FIO_2 _(> 0.8) ventilation > 7 days.^13^.

• Intra-cranial bleeding.

• Any other contra-indication to limited heparinisation.

• Patients who are moribund and have any contra-indication to continuation of active treatment.

Moribund patients are those who the duty ECMO consultant feels have a very low chance of meaningful survival with ECMO treatment.

### Allocation of patients

Selection bias at entry will be minimised by the procedures described below and shown schematically in Figure 1. Potentially eligible patients may be entered into the trial from any participating intensive care unit in the UK. (If a hospital has not yet received ethics committee approval, patients can be entered under an Emergency Inclusion Protocol (EIP)). The referring intensivist will contact a member of the clinical advisory team to confirm that the patient is eligible for the trial, and that beds for ECMO and conventional management are available. These beds will then be 'held' for at least two hours. If these conditions are met, the referring intensivist will discuss the trial with the patient's relative(s), give written information, and ask for agreement to trial entry. The relative will be asked to sign the assent form indicating that he/she believes his/her relative would not object to taking part in the study. The intensivist will then speak to the advisory team and, if the assent procedure has been completed, the advisor will telephone the independent central randomisation service to register the identifying details, and to give information about key prognostic factors. Randomisation will then be to conventional management or to consideration of ECMO support.

Minimization criteria will be used to ensure a balance of key prognostic factors between groups using the following criteria:-

Type of centre (CTC or RH)

Age (18–30, 31–45, 46–65)

Hours of high pressure and/or high FIO_2 _ventilation (0–48, 49–168)

Mode of trial entry (i.e. hypoxic/hypercarbic)

Diagnostic group (pneumonia, obstetric acute respiratory distress syndrome (ARDS), trauma including surgery within previous 24 hours, other ARDS, and other)

Numbers of organs failed 1–2 or 3 or more, failure being defined as an individual SOFA score for that organ of ≥ 2)[17,18].

If a patient is referred into the trial when there is no intensive care unit (ICU) or ECMO bed available that patient will not be entered. If beds become available subsequently, the patient is still suitable and the referring intensivist still wants to enter the patient then they will be randomised in the normal fashion. The fact that these patients were referred but were unable to be entered will be recorded.

### Referrals for trial entry from hospitals not registered as trial centres; Emergency Inclusion Protocol (EIP)

During the study period ECMO will not be offered outside the framework of the trial to patients eligible for trial entry. If, exceptionally, a UK hospital from outside the study wishes to refer a patient, the transport team from the ECMO centre will go to the hospital and assess the patient. If the patient is suitable then they will call the central randomisation service and the patient will be randomised in the normal fashion. If the patient draws conventional treatment, the ECMO team will transport the patient to the nearest available CTC, and if selected for ECMO they will transport the patient back to Glenfield hospital.

### Interventions

#### 1. Conventional management

Patients randomised to conventional ventilatory support will receive the intensive care provided as standard in one of a number of participating CTCs. This may occasionally involve transfer (see Transport, below) from an RH. Conventional ventilatory support can include any treatment modality thought appropriate by the patient's intensivist (excluding ECMO or other extracorporeal techniques). Intensivists will have full discretion to treat patients as they think appropriate. It will be recommended that intensivists adopt the low volume ventilation strategy. Adherence to this strategy is defined for the purposes of CESAR as a plateau pressure <30 cm H_2_O (or if plateau pressure is not measured the peak inspiratory pressure). This will usually mean a tidal volume of 4–8 ml/kg body weight as defined in the low tidal volume ventilation strategy according to the ARDS Network group [19].

Each CTC will produce their own statement of the general philosophy of treatment. This will be based on a pro-forma, which will detail their approach to ventilation, nutrition, antibiotics and other treatment issues. This pro-forma will also collect basic data regarding the size of unit, number of staff, cases treated per year etc.

#### 2. ECMO

Patients randomised to ECMO will be transferred (see Transport, below) to the ECMO centre for consideration of ECMO support. During the trial, adult ECMO will only be available as part of the trial. There will be no crossover to ECMO for patients allocated to conventional management. ECMO will be provided according to published Glenfield Hospital treatment protocols[9]. This protocol is very similar to those used in other ELSO recognised adult ECMO centres [14], and is summarised below:

Veno-venous ECMO via percutaneous cannulation is used if the patient's haemodynamic status is sufficiently stable to make cardiac assist (via veno-arterial access) unnecessary. Blood is drained from the right atrium through a cannula introduced via the right jugular or femoral veins, and is returned via the contra-lateral femoral vein. Circuits are designed to allow full support of gas exchange i.e. blood flow of 120 ml/kg/min. One or two (depending on body weight) Medos Hi-Lite 7000LT poly-methyl pentene lungs with heat exchangers are arranged in parallel with counter current gas flow, 100% oxygen is used as the sweep gas. Stockert (Sorin Biomedical) roller pumps with bladder box servo control or venous pressure servo-regulation are used. Blood raceway tubing is Tygon S-65-HL (Norton Performance Plastics). Normothermia is maintained. The circuit and patient are managed 24 hours per day by a trained "ECMO Specialist" capable of performing surveillance and emergency repairs to the circuit.

During ECMO, ventilator settings are gradually reduced to allow lung rest, i.e. peak inspiratory pressure 20 cm H_2_O, end expiratory pressure 10 cm H_2_O, rate 10 breaths per minute and FIO_2 _30%. Anticoagulation is maintained with heparin to keep the activated clotting time (ACT) between 160 and 220 seconds. Patients are fed enterally or parenterally into the circuit, as indicated. Invasive procedures are avoided to reduce the risk of haemorrhage, and therefore any additional venous access necessary, e.g. for haemofiltration, is achieved via the circuit. Patients are diuresed to dry weight. Haemoglobin concentrations are maintained at 14 g/dl, and platelet counts are kept >100,000 per ml. Patients are weaned from ECMO and decannulated when chest X-ray appearance and lung compliance have improved, and adequate gas exchange without excessive ventilation (peak pressure less than 30 cmH_2_O, and FIO_2 _less than 60%) can be demonstrated during a 'trial-off' ECMO.

Patients developing liver failure either during or after ECMO (defined as a serum bilirubin >200 uMol/L) are supported with MARS (Molecular Absorbent Recirculating System, Teraklin GMBH, Rostock, Germany).

If the patient's condition alters such that ECMO is no longer possible or appropriate then ECMO will not be initiated. However such a patient's outcome will be analysed as part of the ECMO group (intention to treat).

#### 3. Transport

Patients who are in a designated CTC will not need to be transported if they are randomised to conventional management. All other trial patients will need transport, which will be provided by a team from the ECMO centre. If the transport team decides that it is not safe to move the patient then s/he will remain in the original unit until s/he is considered safe to transfer, or recovers or dies. Such outcomes will also be analysed as part of the treatment option to which the patient was randomised i.e. analysis is by intention to treat.

### Outcome measures

#### Primary

Death or severe disability at six months (defined as death by 6 months or before discharge from hospital at any time to end of data collection, or where the answer to the first two questions of the Euroqol questionnaire (EQ5D) are 'confined to bed' and 'unable to wash or dress yourself').

#### Secondary

I) Hospital indices: duration of ventilation, use of high frequency/oscillation/jet ventilation, use of nitric oxide, prone positioning, use of steroids, length of ICU stay, length of hospital stay. Some data will be recorded daily (see "Economic issues", below). For ECMO patients only, data will be collected on mode (VV/VA), duration of ECMO, blood flow and sweep flow.

II) Health status 6 months after randomisation. This will include activities of daily living, quality of life, respiratory symptoms, cognitive psychological state and lung function. Where applicable carer strain will also be assessed. (see also 'economic issues' below)

III) Surviving patients will be asked to give agreement for information to be held by the NHS Central Register if appropriate, further funding may be requested later for longer-term follow-up including lung function tests.

#### Six month follow-up

Assessment of outcome at the 6 month follow-up will be performed by trained researchers who will interview and examine patients in their homes. Patients and their relatives will be instructed not to reveal which treatment was used. Patients will wear a special scarf to cover the neck, masking the presence or absence of cannulation wounds. The assessment will include a generic measure of health status (SF36[20]) and quality of life (Euroqol EQ5D[21]), respiratory related quality of life (St George's Hospital Respiratory Questionnaire[22]), psychological state (Hospital Anxiety and Depression Scale[23]) and cognitive function (Mini-Mental State Examination[24]). The interview will also include specific questions on sleep (from the Functional limitation profile[25]). Lung function will be assessed by spirometry. Where applicable, effects on the carer will be measured using the carer strain index[26]. If a home visit is unacceptable, patients will be offered a telephone interview or postal questionnaire. For those unwilling to be assessed by interview or questionnaire, permission will be requested for information to be sought from the patient's general practitioner.

### Longer term follow-up

Further follow up will be the subject of a separate protocol. So that the study organisers do not lose contact with patients should they move addresses, and also to follow up on health status, patients are being asked to give their agreement for their contact details to be sent to the NHS Central Register.

### Economic issues

The primary objective of the economic evaluation is to assess incremental cost-effectiveness of ECMO in terms of additional survival with and without disability at six months post-randomisation. This will be done by determining the costs to health services and households, assessing cost-effectiveness from the viewpoint of the NHS and also from the societal viewpoint. The overall approach will be to describe the care received by patients in both arms of the trial, identifying use of health services with potentially important costs or changes in household resources.

The trial will assess the cost of treatment to the health and social services and to patients and their families in each treatment group. An incremental cost-effectiveness ratio will be calculated and compared to that for similar life-extending treatments. Information for the costs of inpatient and domiciliary care will be collected using methods adapted from the neonatal ECMO Trial [21-23]

Costs of care will be estimated by recording use of key health care services as part of the data set for each person in the trial, and separately estimating costs associated with each item of health care use. Service use will be measured as daily level of intensive care support, until discharge to an ordinary ward. Subsequent health care costs will be based on days of inpatient care, and use of transport, outpatient and primary care services. Resource use after discharge from hospital will be collected by questionnaire at 6 month follow up. After discharge home, trial participants will be sent an 'aide memoire' to record health service contacts.

Societal costs will be estimated for this trial as the net total costs to health services and to patients. Societal costs of illness can also include the costs borne by relatives and friends of visiting, supporting and caring for the patient. It is likely that visiting costs will differ between trial arms. A literature review found no studies of visiting costs for adult patients. A pilot study conducted outside the CESAR trial has established a survey method for measuring costs[24] and will be conducted in a sub-sample of ICUs taking part in the trial and willing to do the additional research, in order to describe typical visiting costs for patients in ECMO and conventional centres.

To estimate levels of intensive care, data will be collected within the trial about the nature and duration of organ system support for individual patients. Data will be collected at the same time as the trial from participating intensive care centres and the ECMO centre to estimate costs of each level of care using a standard methodology [25,26]. Health care service use after discharge will be derived from a questionnaire to patients at 6 months. Patients agreeing to participate will be invited to complete a simple diary as a memory aid to assist completion of the 6-month questionnaire. Household costs will be determined according to any changes the patients may have experienced in household circumstances (including major costs related to the illness and changes in economic activities).

Cost-effectiveness in terms of disability free survival and quality-adjusted life years gained will be estimated based on 6-month responses to the Euroqol EQ5D questionnaire.

Finally, the implications of the trial for efficient provision of ECMO services in the UK will be considered. Until the end of the trial, ECMO will only be available in one centre. Cost analysis will be done to assess sensitivity of cost-effectiveness ratios to transport and local volume of service in the ICU and ECMO unit in order to predict the best configuration of ECMO services, if the treatment is effective.

#### Data collection instruments for economic evaluation

##### 1) For trial patients and relatives

a) Daily organ support chart to be completed by caregivers in intensive care units for each patient in the trial

b) Patient's diary of events after discharge – to be completed and kept by patient to help answer questions at 6 months.

c) EQ5D health related quality of life questionnaire

d) Patient's and relative's costs questionnaire: versions for survivors, and for relatives of patients who die (self completed)

##### 2) For participating centres

a) ICU cost estimates derived from a national DH funded study conducted by one of the trial investigators [27,28] for each ICU (and equivalent for ECMO centre during final year of trial)

b) Daily ward costs from participating hospitals (based on finance data)

c) Transport costs

Other health and social care unit costs will be based on nationally available data (e.g. Netten and Dennett, PSSRU, University of Kent 1999 or NHS reference costs) or special costing exercises by researchers.

### Sample size

A 70% mortality in the control group is anticipated, based on the NIH ARDS network database. Cross-referencing with the Case Mix Programme Database, which is the national comparative audit of patient outcomes co-ordinated by the Intensive Care National Audit & Research Centre (ICNARC) confirms that this estimated mortality is approximately correct. The mortality of the 1,506 patients with a PaO_2_/FIO_2 _ratio of ≤ 100 mmHg in this database was 61.6%. The mean PaO_2_/FIO_2 _ratio in the ECMO patients was 65 mmHg with an SD of 37. Thus the selection criteria of a Murray score of = 3.0 should successfully identify patients with an expected mortality of = 70%. In addition this is also the patient group that is currently receiving ECMO. Assuming a 10% risk of severe disability among survivors in both trial arms, an alpha = 0.05 (2 sided test) and beta = 0.2, a sample size of 120 patients in each group (i.e. a total sample size of 240) would be required to detect a reduction in the rate of primary outcome from 73% to the 55% which is a conservative estimate based on the descriptive studies of adult ECMO already discussed. As there is some controversy about the estimated mortality in the control group, a power calculation grid is included for a range of estimated mortalities (Table 1), should data from the on-going trial suggest a different level. The sample size was reviewed June 2003 when the Principal Investigators made anapplication for an extension of funding to the Health Technology Assessment Programme (HTA). In the original application, they provided a grid showing the implications of different estimates for the primary outcome in the control group and for the size of difference. This showed, for instance, that with a sample size of about 240 if the primary outcome rate in the control group was about 57% or more they would be able to detect a reduction by a third OR if the primary outcome rate in the control group was about 73% or more, they would be able to detect a reduction by a quarter. If the primary outcome rate in the control group was around 65% or more, a sample size of about 180 would allow them to detect a reduction by a third (all estimates based on 5% statistical significance (2-sided test) and 80% power). The HTA agreed an extension of recruitment by which time CESAR is likely to recruit about 180 patients.

### Recruitment rate

Glenfield ECMO unit treated 40–50 adults per year (prior to 2001). In 1997, 28 hospitals referred 44 patients for ECMO. If all 224 Intensive Care Units (ICUs) in the UK hospitals were to refer patients for ECMO at the same rate as the 28, a total of around 350 patients might be eligible for trial entry in the UK per annum. It is unlikely that all 224 centres will participate, so some patients will be treated in hospitals not participating in the trial and some will not be asked for nor give assent for the trial. If 100 centres do wish to take part, it should be possible to recruit sufficient patients over the recruitment period.

### Statistical analysis

#### Type of analysis

Analysis will be by intention to treat, with sub-group analyses based on the minimisation criteria at trial entry.

#### Frequency of analysis

An independent Data Monitoring Committee (DMC) will review, in strict confidence, data from the trial approximately half way through the recruitment period. The Chair of the DMC may also request additional meetings/analyses. In the light of these data, and other evidence from relevant studies, the DMC will inform the Steering Committee, if in their view:

i) there is proof beyond reasonable doubt that the data indicate that any part of the protocol under investigation is either clearly indicated or contra-indicated, either for all patients or for a particular subgroup, or

ii) it is evident that no clear outcome will be obtained with the current trial design.

Unless modification or cessation of the protocol is recommended by the DMC, the Steering Committee, collaborators and administrative staff (except those who supply the confidential information) will remain ignorant of the results of the interim analysis.

#### Membership of Data Monitoring Committee

Professor Sir Richard Doll (Chair until 2005), Professor Douglas Altman (Chair from 2005), Professor Tim Evans and Dr Duncan Macrae.

### Ethical considerations

Since the patients in this trial will all be sedated and ventilated the patient's next of kin will be asked to give assent for the patient's inclusion in the trial. There will be information booklets for the patient's relatives which will include information about the trial, conventional treatment and ECMO. This may raise some ethical issues since strictly speaking the patient's next of kin can only assent for treatment of an incompetent adult, and cannot give true consent on their behalf. However, there is a duty of care to act in the patient's best interests and apply whatever treatment is believed to be the most effective. Since in this case it is not yet clear which treatment is most effective there is a larger duty of care to the community as a whole to determine which treatment is most effective by means of an RCT. When patients have recovered and been discharged home they will be informed that they have been part of a clinical trial and given a copy of the information leaflet. During the trial period patients who would be eligible for the trial will not be able to get ECMO in the UK except as part of the trial.

The trial has been approved by the Trent Multicentre Research Ethics Committee (REC) as well as relevant Local RECs,

### Ancillary studies

In addition to addressing the main aims of the study, some collaborators may wish to conduct other more detailed or complementary ancillary studies. The principal investigators welcome this provided that proposals are discussed in advance with the Trial Steering Committee.

### Publication policy

To safeguard the scientific integrity of the trial, data from this study should not be presented in public or submitted for publication without requesting comments and receiving agreement from the Trial Steering Committee. The primary results of the trial will be published by the group as a whole although the paper will be written by a smaller writing committee, and a table of contributors will delineate individual investigators' personal contributions to the study. The success of the trial depends on the collaboration of many people.

### Organisation

#### Principal investigators

i) *Giles Peek*: Will co-ordinate the activities of the collaborators at all clinical centres and the project staff at Glenfield Hospital Leicester, the Clinical Co-ordinating Centre, will organise the clinical advisory service and in conjunction with the clinical research fellow will promote the trial to encourage participation of referring centres. Will be closely involved in data analysis and a key member of the writing committee.

ii) *Diana Elbourne: *Will co-ordinate activity at the London School of Hygiene and Tropical Medicine (LSHTM), the Data Co-ordinating Centre with particular responsibility for data collection, management and statistics. Key member of writing committee, senior statistician.

iii) *Richard Firmin: *Will work closely with Giles Peek and will be head of the clinical advisory service.

iv) *Ann Truesdale: *Will work closely with Diana Elbourne as Study Co-ordinator working with staff at the LSHTM and form part of the writing committee.

v) *Miranda Mugford: *Will co-ordinate the economic study team and work closely with Clare Hibbert, and form part of the writing committee.

vi) *Hilliary Killer: *Will assist in the day to day management of the trial at the ECMO centre and will work closely with the economic study team. Will form part of the clinical advisory team. Will provide a nursing and technical viewpoint.

vii) *Clare Hibbert: *Will be a member of the economic study team with Miranda Mugford.

viii) *Andy Wilson: *Will co-ordinate the activities of the GP Advisory Group and take responsibility for the follow-up assessment at six months and form part of the writing committee.

### Trial Steering Committee

The Steering Committee will approve the main study protocol, monitor and supervise the trial towards its interim and overall objectives, review relevant information from other sources, consider the recommendations of the DMC, and resolve problems brought by the trial co-ordinating centres. The committee will comprise an independent chairperson, Professor David Field, independent members, Ms Jayne Fawcett(University of York), Dr David Goldhill (Consultant Anaesthetist, Royal National Orthopaedic Hospital), Mrs Silvia Holden (Cruse Bereavement Care), Mrs Wendy Nganasurian (Patients Association), Professor Anne Tattersfield (Professor of Respiratory Medicine, Nottingham City Hospital), Dr John Scott (East Anglian Ambulance Trust) Professor Nigel Webster (Professor of Anaesthesia and Intensive Care, Aberdeen Royal Infirmary)as well as the members of the project management group. This represents all the different disciplines involved in the trial. Specialist working groups will advise the Steering Committee.

### Project Management Group (PMG)

A project management group will be established and will be responsible for the day to day management of the trial. The group will comprise the principal investigators and project staff from the Clinical Co-ordinating Centre at Leicester and the Data Co-ordinating Centre at the LSHTM and from the health economics group based at UEA Norwich and School of Health and Related Research (ScHARR) in Sheffield. The group will meet regularly in person and by telephone.

The responsibilities of the PMG include:

a) Establishing and monitoring recruitment of participating centres

b) Distribution and supply of data collection forms and other appropriate documentation for the trial

c) Data collection and management

d) Data entry and cleaning

e) Data analysis

f) Organising and servicing the Data Monitoring Committee

Local co-ordination

Each participating centre will identify an intensivist as a local co-ordinator and two intensive care nurses (one primary and one as backup).

The responsibility of the local co-ordinators will be to:

a) Ensure local research ethics approval is obtained

b) Be familiar with the trial and consider recruitment of potentially eligible patients

c) Liaise with the Clinical Co-ordinating Centre to register eligible patients

d) Liaise with the transport team when relevant

e) Liaise with the Data Co-ordinating Centre

f) Ensure that relevant medical and nursing staff are informed about the trial

g) Ensure that mechanisms for recruitment are in place

h) Ensure that data collection forms are completed and returned to the Data Co-ordinating Centre promptly and to deal with any queries

i) Facilitate other aspects of co-ordination as relevant

j) Make data available for verification, audit and inspection purposes as necessary

k) Liaise with the economics team

l) Ensure that the confidentiality of all information about trial participants is respected by all persons

### Confidentiality

Patients will be identified by their trial number to ensure confidentiality. However, as the patients in the trial will be followed up to 6 months following randomisation, it is essential that the team at the Data Co-ordinating Centre has the names and addresses of the trial participants recorded on the data collection forms in addition to the allocated trial number. Stringent precautions will be taken to ensure confidentiality of names and addresses at the Data Co-ordinating Centre. The investigators and local co-ordinators will ensure conservation of records in areas to which access is restricted.

## Discussion

The CESAR Trial should define the appropriate use of extra-corporeal life support for adults with severe potentially reversible respiratory failure. It will also determine the cost efficacy of such treatment. CESAR will also provide profound insight into the conventional treatment of such patients in the UK.

## Abbreviations

ARDS Acute Respiratory Distress Syndrome

CESAR Conventional Ventilation or ECMO for Severe Adult Respiratory Failure

cmH2O Centimetre of water

CTC Conventional Treatment Centre

CXR Chest X-Ray

DH Department of Health

DMC Data Monitoring Committee

ECCO2R Extracorporeal Carbon Dioxide Removal

ECMO Extracorporeal membrane oxygenation

EIP Emergency Inclusion Protocol

ELSO Extracorporeal Life Support Organization

EQ5D Euroqol questionnaire

FIO2 Fraction of inspired oxygen

ICNARC Intensive Care National Audit and Research Centre

ICU Intensive Care Unit

IPPV Intermittent Positive Pressure Ventilation

KPa Kilopascals

LSHTM London School of Hygiene & Tropical Medicine

MARS Molecular adsorbent recirculating system

mmHg Millimetres of mercury

NIH National Institute of Health

NSCAG National Specialist Commissioning Advisory Group

PaO2 Partial pressure of oxygen in arterial blood

PCIRV Pressure Controlled Inverse Ratio Ventilation

PEEP Positive End Expiratory Pressure

pH negative base 10 logarithm of the hydrogen ion concentration in millimoles per litre

PIP Peak Inspiratory Pressure

RCT Randomised Controlled Trial

RH Referral Hospital

ScHARR School of Health and Related Research

SERNIP Safety & efficacy register of new interventional procedures

SF36 Short form 36 questionnaire

SOFA Sequential Organ Failure Assessment

TV Tidal Volume

UEA University of East Anglia

UK The United Kingdon of Great Britain and Northern Ireland

VA Veno-Arterial

VV Veno-Venous

## Competing interests

GJP, RKF, HMK and RT are all clinicians involved in ECMO

## Authors' contributions

GJP conceived the study. GJP, DE, RKF, CH, HMK, MM and AT were applicants for the funding. All authors were involved in designing the study and drafting the protocol. All authors read and approved the final protocol.

## Appendix 1: Murray score

The Murray score is a grading system for ARDS which uses 4 pieces of information graded 0–4 to give a severity index for ARDS. The data required are:

• PaO_2_/FIO_2 _in mmHg (multiply Kpa result × 7.5): this must be taken with the FIO2 at 1 for at least 20 minutes.

• PEEP in CMH_2_O

• Lung Compliance in ml/CMH_2_O

• Number of quadrants with infiltration seen on chest X-ray

Patients can be registered for the trial when the Murray Score exceeds 2.5, and are eligible to enter and be randomised when it exceeds 3.0. Patients who are hypercarbic, but not hypoxic and therefore have a low Murray score may enter the trial and be randomised once the arterial pH falls below 7.2. The Murray score is calculated by taking the score for each variable and dividing by 4, for the purposes of the CESAR trial all 4 variables must be used to calculate the score.

### Score values

• PaO_2_/FIO_2_: ≥ 300 = 0, 225–299 = 1, 175–224 = 2, 100–174 = 3, <100 = 4

• CXR: normal = 0, 1 point per quadrant infiltrated.

• PEEP: ≤ 5 = 0, 6–8 = 1, 9–11 = 2, 12–14 = 3, ≥ 15 = 4.

• Compliance (ml/cmH_2_O): ≥ 80 = 0, 60–79 = 1, 40–59 = 2, 20–39 = 3, and ≤ 19 = 4

The compliance may be calculated as follows: TVPIP-PEEP
 MathType@MTEF@5@5@+=feaafiart1ev1aaatCvAUfKttLearuWrP9MDH5MBPbIqV92AaeXatLxBI9gBaebbnrfifHhDYfgasaacH8akY=wiFfYdH8Gipec8Eeeu0xXdbba9frFj0=OqFfea0dXdd9vqai=hGuQ8kuc9pgc9s8qqaq=dirpe0xb9q8qiLsFr0=vr0=vr0dc8meaabaqaciaacaGaaeqabaqabeGadaaakeaadaWcaaqaaiabbsfaujabbAfawbqaaiabbcfaqjabbMeajjabbcfaqjabb2caTiabbcfaqjabbweafjabbweafjabbcfaqbaaaaa@37D6@

where TV is Tidal Volume, and PIP is Peak Inspiratory Pressure

Example

• A patient has a PaO_2 _of 6.6 Kpa on 100% oxygen: To convert KPa to mmHg = 6.6 × 7.5 = 49.5 mmHg, divide by the FIO_2 _( = 1), the PaO2/FIO2 is 49.5, as this is less than 100, **score 4**

• The Chest X-ray has consolidation and infiltration in 3 out of 4 quadrants **score 3**

• The PEEP is set at 10 CMH_2_O, **score 2**

• The Peak airway pressure is 38 CMH_2_0, and the tidal volume is 420 ml, PIP-PEEP = 28, compliance is 420/28 = 15, **score 4**

The Murray score is (to one decimal place):

4 + 3 + 2 + 4 = 13,

13/4 = **3.3**

The Murray score is high enough for trial entry (>3)

## Pre-publication history

The pre-publication history for this paper can be accessed here:



## Figures and Tables

**Table 1:** POWER CALCULATION GRID

		Mortality in Control Group											
		
		70%			60%			50%			45%		
% of survivors severely disabled		15%	10%	5%	15%	10%	5%	15%	10%	5%	15%	10%	5%

	Primary adverse outcome %	74.5	73	71.5	66	64	62	57.5	55	52.5	53.25	50.5	47.75

Relative risk	0.5	64	66	70	82	86	92	104	112	122	118	128	140
	0.67	136	142	150	**180**	192	204	236	**256**	**278**	**270**	**296**	**326**
	0.75	224	236	**250**	**302**	**324**	**348**	**404**	**440**	**480**	**468**	**514**	**566**
	0.8	**336**	**356**	**378**	**462**	**496**	**532**	**624**	**682**	**746**	**726**	**800**	**62**

Sample size calculation for different assumptions about mortality, disability and relative risk (Beta = 0.2, Alpha = 0.05, 2 sided)
